# Editorial for “Special Abilities of Microbes and Their Application in Agro-Biology”

**DOI:** 10.3390/microorganisms12061179

**Published:** 2024-06-11

**Authors:** Anuj Ranjan, Vishnu D. Rajput, Abhishek Chauhan

**Affiliations:** 1 Academy of Biology and Biotechnology, Southern Federal University, Stachki 194/1, 344090 Rostov-on-Don, Russia; rvishnu@sfedu.ru; 2 Amity Institute of Environmental Toxicology, Safety and Management, Amity University, Noida 201301, India; akchauhan@amity.edu

Currently, climate change-related environmental issues associated with agriculture is alarming and poses a potential risk to global food security, causing significant global concern as a result [1]. Achieving the United Nations’ Sustainable Development Goal of “Zero Hunger” necessitates the collective pursuit of sustainable agricultural practices [2]. Among these practices, the use of microbial-based biofertilizer has gained significant popularity due to its multifaceted role in agriculture. Microbes thriving in various environments are adapted and equipped with unique physiological and metabolic functionalities, which, upon their harnessing, could be useful for sustainable agricultural practices [3]. For instance, psychrophiles possessing plant growth-promoting traits could be useful for promoting soil fertility and crop production in geographically colder regions [4]. Similarly, thermophiles can be employed in a comparative tropical region where draught and soil–water stress are evident [5]. Therefore, this Special Issue, titled “Special Abilities of Microbes and Their Application in Agro-Biology”, concerns the interaction between plants and beneficial microbes, and its scope covers the development of possibilities for enhancing agricultural productivity while mitigating environmental stressors. The studies reported in this editorial reveal the potential of beneficial microbes in agriculture.

Considering their evident adaptability to harsh environments and the usefulness of this special ability, one study on microbes in agriculture reports on anthropogenically polluted soil harboring microbes that have evolved the ability to survive, hence being useful for agricultural purposes, as exemplified by the species of *Bacillus*, *Brevibacterium*, and *Pseudomonad*, which exhibited exceptional plant growth-promoting traits [6]. Resilience to copper (Cu)-induced stress and the strengthening of the resilience of *Brassica napus* L. have been witnessed by using the metallotolerant *Bacillus altitudinis* strain TF16a, where Cu treatment was found to be accumulated in the roots and shoots and also elevated malondialdehyde (MDA) content by 20%. The application of a biofertilizer (prepared with biochar and *B. altitudinis* strain TF16a) with Cu decreased its accumulation by 20% for the shoots and 28% for the roots, maintaining MDA content similar to the control group, and both biofertilizer treatments, with and without Cu, increased chlorophyll a and b content, as well as non-enzymatic antioxidants, and led to increased biomass in the shoots and roots [7]. Improved phosphate mineralization and ex vitro acclimatization of *Musa acuminata* var. Valery using *Rahnella aquatilis* AZO16M2 was also evident when using microbes as a PGPR. In a solid medium with Ca_3_(PO_4_)_2_, *R. aquatilis* AZO16M2 exhibited a solubilization index (SI) of 3.77 at 28 °C, and in a liquid medium, it produced 29.6 mg/L of soluble P at a pH of 4.4 [8].

In addition to this topic, this Special Issue attempts to provide details on microbial ecology, where the dynamics of soil microbiota come to light, such as in a case in which endophytic bacteria *Enterobacter* sp. ABk36 and HSTU-ABk39 were found to mineralize chlorpyrifos and support the health and growth of rice [9]. Similarly, phyllospheric bacteria isolated from *Coffea arabica* were found to be effective against coffee rust. The isolates CRRFLT7 and TRFLT8 showed urediniospore germination inhibition at rates of 81% and 82% [10], demonstrating the impact of microbial symbiosis in the protection of crop health and enhancing agricultural productivity. Through the transformative power of solid-state fermentation (SSF), another review article discusses approaches to unlock the underlying potential of waste materials to yield value-added agricultural formulations such as bio-stimulants and biopesticides [11], and it presents an evaluation of enhanced Brewer’s spent grain’s nutritional value and safety for use in animal feed [12]. By harnessing the principles of the circular economy, such innovative approaches promise to revolutionize agricultural production while minimizing environmental impact, heralding a new era of sustainable agriculture.

Additionally, this Special Issue includes an article that concerns precision agriculture, where nanotechnology emerges as a tool in the mitigation of plant pathogens and environmental stressors. Through the utilization of nano-minerals as alternatives to chemical fungicides, the researchers of the article demonstrated that the mycelial growth of *A. alternata* was inhibited by 85.1% with the application of 100 ppm nano-Se and that combining Se with SiO_2_ at half doses resulted in a slightly lower efficacy rate of 77.8%, which could be significantly useful for crop protection strategies in sustainable crop management [13]. Lastly, this Special Issue also concerns ecosystem management, where a balance between pollinators, pathogens, and agricultural ecosystems comes into focus. In a separate study, the impact of probiotics on honeybees fed with a probiotic EM^®^ for bees (TH2; TH3) showed a significant reduction in pathogen *Nosema* spp. spore counts from 25.18% to 96% on average across different sampling days, while the control groups (TH1, TH4) exhibited a continuous increase in infection levels along with improvement in gut microbiota [14]. To unravel the interactions between plants, diseases, and arthropods in open-field conditions, another study highlighted the impact of *Trichoderma harzianum* T22 on zucchini plants in open-field conditions, revealing increased attractiveness to aphids and *Hymenoptera parasitoids* but ineffectiveness against zucchini pathogens [15]. Similarly, a different study assessed the sensitivity of *Plasmopara halstedii* isolates to mefenoxam in sunflowers by analyzing host responses, including disease severity, growth reduction, and tissue reactions [16], to support biodiversity and the adoption of ecological resilience, which is the foundation of a more sustainable and resilient agroecosystem for the future.

In conclusion, this Special Issue stands as evidence of the potential of microbes with special abilities for utilization in innovative and collaborative efforts that address the multifaceted challenges facing modern agriculture. As we come across the complexities of rapidly changing climatic conditions, such collective endeavors bring us closer to a future where agriculture can thrive in harmony with nature.
